# Planting the Seed of Epistemic Curiosity: The Role of the Satisfaction of the Needs for Autonomy and Competence

**DOI:** 10.3390/jintelligence12120127

**Published:** 2024-12-13

**Authors:** Lizbeth Puerta-Sierra, Rogelio Puente-Díaz

**Affiliations:** School of Business and Economics, Universidad Anahuac México, Av. Universidad Anáhuac 46, Huixquilucan C.P. 52786, Mexico; lizbeth.puerta@anahuac.mx

**Keywords:** autonomy, competence, epistemic curiosity, entrepreneurial mindset, epistemic activities, learning

## Abstract

We posit that curiosity, an epistemic emotion, is an integral part of entrepreneurial education, entrepreneurial activities, and learning, which seek to develop an entrepreneurial mindset, a form of cognitive ability. The purpose of the present investigation was to examine the influence of autonomy and competence satisfaction on epistemic curiosity and the influence of epistemic curiosity on epistemic satisfaction and performance on epistemic activities in entrepreneurial education. Participants completed a battery of questionnaires in three waves of data collection. Results showed a positive relationship between the satisfaction of the need for autonomy and competence in times 1 and 2. The satisfaction of the need for competence at times 1 and 2 then had a positive relationship with epistemic curiosity at times 1 and 2. Curiosity at time 2 had a positive relationship with epistemic satisfaction at time 3. Our results supported the idea that autonomy and competence satisfaction facilitated the experience of epistemic curiosity and epistemic satisfaction. The implications of developing an entrepreneurial mindset were discussed.

## 1. Introduction

Curiosity, defined as the desire to gather and acquire new information (Berlyne 1954), is an integral part and a catalyst of learning (Gruber et al. 2014), especially lifelong learning (Muis et al. 2018). Entrepreneurial education values learning, learning about the category where students want to innovate, and learning about the process of seizing and creating new business opportunities (Puerta-Sierra and Puente-Díaz 2023). Without learning, students cannot generate entrepreneurial ideas. Without lifelong learning, business ideas cannot be modified or adjusted to specific environmental demands. Hence, we posit that curiosity is essential for learning in entrepreneurship education. Curiosity is essential to build an entrepreneurial mindset. One of the priorities of entrepreneurial education should be to “plant the seed of curiosity”. Internal and external factors mold students’ curiosity to learn. Among the external factors, professors play an important role in the process of learning in entrepreneurial education, not only because of the knowledge they teach but also because of the “seed of curiosity” that they might be able to plant in their students. Specifically, professors, with their teaching and motivational styles, could help students feel autonomous and competent in their entrepreneurial education with adaptive affective and behavioral consequences. Thus, the purpose of this study is to examine the relationships between the satisfaction of the need for autonomy and competence and curiosity. In addition, we examine how the satisfaction of the need for autonomy and competence, and curiosity relate to epistemic satisfaction and performance on epistemic activities among entrepreneurship students. Our study seeks to make a small contribution by suggesting that one of the main objectives of entrepreneurial education is to plant the seed of curiosity. To achieve our two objectives, we first discuss the educational entrepreneurial environment, followed by the theoretical development of need satisfaction and curiosity, and a brief discussion of epistemic satisfaction and activities.

### The Educational Entrepreneurial Environment

Entrepreneurship is a life skill for the 21st century (Neck and Corbet 2018). Entrepreneurship education teaches students to be better at creativity, team building, resource acquisition, and finding ways to mitigate social problems. It focuses on developing mindsets, skills, and practices for building new firms (Neck and Corbet 2018). Experiential entrepreneurial education represents an action-based pedagogy to introduce students to the field of entrepreneurship (Lackéus 2020) and encourage students to be active learners. This experience requires students’ engagement in the process of venture creation (Pazos et al. 2022). Venture creation programs in entrepreneurship education must not only focus on creating a venture (Haneberg et al. 2022), but also on the process of discovering opportunities, business ideation, and business planning (Pazos et al. 2022), emphasizing a need for continuous, lifelong learning. This form of active learning is often organized around small-scale activities, something referred to as epistemic activities in the current study.

A lifelong learning approach emphasizes constantly exploring what has been learned and understood about experiential entrepreneurial programs (Haneberg et al. 2022). Hence, students need to value the acquisition of knowledge and its constant revision. Students need to develop cognitive and metacognitive skills that allow them to become aware of changes in their levels of learning through external interaction, feedback, and support from people, including professors (Lackéus 2020). It seems that professors’ teaching and motivational styles are likely to play an important role.

In addition, curiosity is among the strongest predictors of an individual´s tendency to start a business, whether it is treated as something that fluctuates, state curiosity, or something more stable, trait curiosity (Heinemann et al. 2022). For this reason, we take the opportunity to join a relevant conversation by assessing the connections between a teaching style that supports the satisfaction of the need for autonomy and competence, and curiosity with their respective consequences. Previous research on entrepreneurship education has not extensively focused on curiosity (Heinemann et al. 2022). Thus, we seek to fill this gap. We now turn to the theoretical bases of our article which includes the discussion of the satisfaction of the need for autonomy and competence and the conceptual developments in curiosity.

## 2. Theoretical Developments

In this section, we briefly discuss self-determination theory (SDT) and the conceptual developments on curiosity to establish the theoretical bases for our empirical investigation. We first discuss SDT.

### 2.1. Self-Determination Theory (SDT)

Given the importance of lifelong learning for entrepreneurship, we need to discuss what might facilitate or hinder this type of learning. Self-determination theory is well-equipped to serve as our guiding framework (Ryan and Deci 2018). Self-determination theory posits that individuals are inherently curious about learning and exploring their environment. Yet, this curiosity could be enhanced or hindered by whether important others, including professors, satisfy the need for autonomy, competence, and relatedness (Ryan and Deci 2018). Autonomy refers to perceiving oneself as the origin of important decisions. Competence refers to perceiving that one is capable of dealing with important challenges in the environment. The satisfaction of both needs, competence and autonomy, has been widely examined in education (Ryan and Deci 2018). Yet, to our knowledge, limited attention has been given to autonomy and competence satisfaction in the context of entrepreneurial education and as an antecedent of curiosity. Most empirical findings indicate that the satisfaction of both needs is important to observe positive, educational outcomes (Ryan and Deci 2018). We seek to make a modest contribution by suggesting that both needs, autonomy, and competence, are needed to energize students’ curiosity. This is especially relevant for entrepreneurship education.

In its theoretical discussion of needs, self-determination theory establishes two propositions that are essential to our conceptual development: (1) The satisfaction of the need for autonomy and competence varies across time and social situations (Ryan and Deci 2018). What this proposition suggests is that individuals actively assess their social contexts and whether they support their need for competence and autonomy. For our study, the direct implication is that students’ perception of the satisfaction of both needs is important and likely to vary leading to either adaptive or non-adaptive outcomes. (2) The satisfaction of the need for autonomy is a prerequisite to the satisfaction of the need for competence (Ryan and Deci 2018). This is so because an environment that supports students’ autonomous decision-making has the potential to facilitate the acquisition of the skills needed to learn and adopt an entrepreneurial mindset. In the process of acquiring the skills needed to learn, students are likely to perceive that their competence is also developing.

In an educational environment, professors are likely to influence students’ perceived autonomy support. We make the claim that the satisfaction of autonomy is necessary for students to feel competent about their ability to generate ideas for new business ventures. Without autonomy, students might not feel capable of generating truly original and effective ideas. Without competence, students would lack the necessary tools to compete in business environments with their ideas for new business ventures. Hence, we first propose the following:

**H1:**  *The satisfaction of the need for autonomy will be positively related to the satisfaction of the need for competence (see Ryan and Deci 2018 for conceptual support).*

One of the positive consequences of feeling autonomous and competent is installing in students the desire to learn. The desire to learn, to develop their personal epistemology (Muis et al. 2018) regarding new business ventures, is driven by curiosity. We turn our discussion to the development of the concept of curiosity.

### 2.2. Curiosity

The conceptual developments of curiosity go back at least 80 years with the initial work on perceptual curiosity (Berlyne 1954). More recent developments (see Litman 2019 for an overview) involve the creation of a conceptual model distinguishing between the type of curiosity energized by a deficit in knowledge referred to as deprivation-type curiosity and the type of curiosity coming from the joy and pleasure of learning, interest-type curiosity. It is a robust model for learning because it integrates situations in which students need and want information with situations in which students would like to have and learn new information. Needing and wanting information implies a deficit, an uncertain situation that needs to be resolved, energized by a wanting-type appetite as a motivational driver (Litman 2005). Liking information implies a more spontaneous, intrinsic type of approach to acquiring and learning new information, energized by a liking-type appetite as a motivational driver (Litman 2005).

Entrepreneurial students are likely to encounter both situations, situations in which they acquire new knowledge for the sake of acquiring, and situations in which they want and need new knowledge. We suggest that the core of entrepreneurial education is planting in students the desire to learn new information for the sake of learning. While it is true that the ultimate goal of entrepreneurial education is to seize business opportunities, it is a long-term, distal goal. A more proximal goal is learning about the basic principles of business, the basic principles of entrepreneurship as a field, the importance of consumers’ needs, and the core components of the category where they would like, later on in this process, to generate better ideas. We claim that the first step in entrepreneurial education is to plant the seed of interest-type curiosity to engage in epistemic activities organized in entrepreneurial education so students can learn. While it is not our purpose to posit and test which of the two types of curiosity leads to more adaptive outcomes in education, it is worth mentioning that interest-type curiosity is positively related to greater tolerance for ambiguity and the unknown, to a more optimistic outlook and greater risk-taking (Litman 2019). All these attributes are adaptive in entrepreneurial projects, especially when students are at the early stages of their entrepreneurial education.

The literature on curiosity also distinguishes between curiosity conceptualized as a trait and as a state (Litman and Spielberger 2003). Given that the focus of our investigation is on examining how curiosity might fluctuate as a function of the satisfaction of the need for autonomy and competence, we will focus on state curiosity and refer to interest-type state curiosity as epistemic curiosity because it comes from engaging in epistemic activities. We briefly review some empirical studies on curiosity in education to show that the examination of the satisfaction of the need for competence and autonomy has not received significant attention.

### 2.3. Empirical Studies

The examination of curiosity in education has received increased attention, yet not as much attention in entrepreneurial education. Thus far, empirical studies have supported the role of curiosity in education and learning. For example, one study found support for the moderating role of curiosity in explaining entrepreneurial passion and intentions (Syed et al. 2020). Similarly, another study found that curiosity played an important role in virtual-reality learning (Ye et al. 2022). Another study found support for the positive influence of curiosity on entrepreneurial intention among diverse samples such as individuals from Saudi Arabia (Elnadi and Gheith 2023). In addition, one study found evidence for the role of epistemic curiosity in predicting intentions beyond the contribution of the personality trait of openness to experience (Heinemann et al. 2022). Most empirical studies have focused on the consequences of curiosity, paying less attention to the antecedents of epistemic curiosity in learning environments. We then seek to address this limitation.

Given that the satisfaction of the need for autonomy and competence facilitates exploration, which is a defining feature of epistemic curiosity, we posit a connection with curiosity. We test the following hypotheses:

**H2:**  *The satisfaction of the need for autonomy will have a positive indirect relationship with epistemic curiosity, through its relationship with the satisfaction of the need for competence (see Ryan and Deci 2018 for conceptual support; Puerta-Sierra and Puente-Díaz 2023 for empirical support).*

**H3:**  *The satisfaction of the need for competence will have a positive direct relationship with epistemic curiosity (see Ryan and Deci 2018 for conceptual support).*

Our proposition is that curiosity is conducive to positive consequences in learning environments (Litman 2005). We focus on two: epistemic satisfaction and performance on epistemic activities.

### 2.4. Consequences of Epistemic Curiosity

Epistemic satisfaction is also an epistemic emotion that provides individuals with information about their epistemic activities (Puente-Díaz and Puerta-Sierra 2024). Epistemic satisfaction implies the fulfillment of one’s wishes and needs in epistemic activities. It is a personal evaluation (Diener 1984). Specifically, we posit that epistemic satisfaction provides individuals with information about their learning experiences with positive consequences such as sustained effort and improved performance (Puente-Díaz 2023; Puente-Díaz and Puerta-Sierra 2024). In addition, scholars have suggested that epistemic emotions such as satisfaction are needed to hold and justify beliefs (Morton 2010), and to aid decision-making (De Sousa 2009). Holding and justifying beliefs is important because students need to evaluate first their ideas to make decisions regarding their potential. Given the important role of satisfaction in epistemic activities, we suggest that curiosity is a driver that could lead to feeling satisfied in learning environments. Consequently, we posit the following hypothesis:

**H4:**  *Epistemic curiosity will have a positive relationship with epistemic satisfaction (see Puerta-Sierra and Puente-Díaz 2023 for empirical support).*

Thus far, we have focused exclusively on epistemic satisfaction, a self-report indicator. We suggest that curiosity could also have behavioral implications. Epistemic activities are integral to active learning methodologies (Lackéus 2020). Epistemic activities are tasks conducted to acquire information. In epistemic activities, students conduct different small-scale activities such as interviewing established entrepreneurs, assessing consumers’ needs, and generating value propositions of their ideas, among others. Epistemic activities seek to enhance active learning and represent a means to an end, in which the ultimate goal is to generate ideas that seize business opportunities. Performance in these epistemic activities is likely to be related to curiosity and epistemic satisfaction. Thus, we propose and test the following hypotheses:

**H5:**  *Epistemic curiosity will have a positive, indirect relationship with performance on epistemic activities (see Puerta-Sierra and Puente-Díaz 2023; Nerantzaki and Efklides 2019 for empirical support).*

**H6:**  *Epistemic satisfaction will have a positive, direct relationship with performance on epistemic activities (see Puente-Díaz et al. 2024 for empirical support).*

In sum, the purpose of the current study is to examine the relationships between the satisfaction of the need for autonomy and competence and epistemic curiosity. The satisfaction of the need for autonomy and competence is treated as an antecedent of epistemic curiosity. In addition, we assess two consequences of epistemic curiosity: epistemic satisfaction and performance on epistemic activities among entrepreneurship students.

## 3. Overview of Study

To design a robust study and overcome the threat of common method bias (Podsakoff et al. 2012), we planned a study with the following features: (1) We collected data in three waves: during the third week of entrepreneurship classes lasting one semester, in the middle, week 8, and the end, weeks 15 and 16, of each class. (2) We included a behavioral component, performance on epistemic activities, as rated by the instructor of each class. Both features should allow us to assess and test with greater precision our research hypotheses.

### 3.1. Method

#### 3.1.1. Participants

Participants were 217 (97% were in the 18–25 age group, 66% females) students enrolled in an entrepreneurship class from [the author has provided it, but it is hidden due to double blindness]. In total, 190 students completed wave 2 and 162 completed wave 3. Entrepreneurship classes were mandatory for all students. Students came from different majors including business, engineering, medicine, design, and the arts, among others. All participants agreed to voluntarily participate in the study. All professors teaching the entrepreneurship classes had similar levels of experience (>five years) and were blind to the research hypotheses. The study was approved by Universidad Anáhuac review board; Approval Code: 243-2023-D and Approval Date: May 2023.

#### 3.1.2. Procedure and Measures

We collected data in three waves. Wave 1 was conducted during the third week of a regular semester class. Participation lasted around 10 min. We decided to collect data on the third week so students could have an idea of the organization of the class, the teaching styles of professors, and the basic information about entrepreneurship. Participants completed a battery of questionnaires assessing autonomy and competence satisfaction, and epistemic curiosity. Students were told that all the questions were in reference to their current experience with the class. Autonomy and competence satisfaction were assessed with the Basic Psychological Need Satisfaction and Frustration Scale (BPNSFS, Van der Kaap-Deeder et al. 2020). The scale assesses autonomy satisfaction and frustration and competence satisfaction and frustration with four items each (sixteen items) on a scale from 1 to 10. Sample items were as follows: “I feel a sense of choice and freedom in the things I undertake (Autonomy support)”, “Most of the things I do feel like I have to (Autonomy frustration)”, “I feel confident that I can do things well (Competence support), “I have serious doubts about whether I can do things well (Competence frustration)”. Total competence and autonomy scores were obtained by subtracting the frustration scores from the satisfaction scores (Van der Kaap-Deeder et al. 2015; α1 = 0.85, α1 = 0.81, respectively). Epistemic curiosity was assessed with four items of the joyful subscale of the Multidimensional Workplace Curiosity Scale (MWCS, Kashdan et al. 2020; α1 = 0.80) on a scale from 1 to 10. Sample items were as follows: “I get excited thinking about experimenting with different ideas” and “I enjoy that I often find my mind continues to work through complex problems outside of school”.

Wave 2 was conducted in the middle of the semester, week 8. Participation lasted around 10 min as in wave 1. During week 8, students had more experience interacting with professors and with the class material. We measured autonomy (α2 = 0.87), competence satisfaction (α2 = 0.88), and curiosity (α2 = 0.75) with the same scales as wave 1. Last, wave three was collected in the last two weeks of the regular semester, weeks 15 and 16. Participation lasted around 8 min. Participants completed the Satisfaction with Life Scale (Diener 1984) modified to assess epistemic satisfaction (α3 = 0.93) on a scale from 1 to 10. Sample items were as follows: “In most ways this class is close to the idea” and “The conditions of this class are excellent”. As part of the class, participants had to conduct several small-scale activities conceptualized as epistemic activities. These activities were graded by professors. The grades ranged from 0 to 10. We considered this indicator as a behavioral component for this study. Examples of activities conducted were as follows: Interviews with entrepreneurs, assessing consumers’ needs with questionnaires, and writing value propositions for their ideas, among others. We used the average grade of these epistemic activities.

## 4. Results

Mplus 7.11 was used to test the latent variable model with a robust estimation to handle missing data and with a bootstrap estimation procedure with 5000 iterations. A combination of absolute and incremental fit indexes was reported: χ^2^, Root Mean Square Error of Approximation (RMSEA), Comparative Fit Index (CFI), and Tucker–Lewis Index (TLI). The χ^2^ has the limitation of being sensitive to the size of the sample. Thus, we will pay greater attention to other indexes. The cutoff scores, as the minimum acceptable levels of model fit, were as follows: RMSEA ≤ 0.08 and CFI and TLI > 0.90 (West et al. 2012). Some methodological scholars suggest having cutoff scores for the CFI and TLI above 0.95. While we do not disagree with the idea of having higher values, we have seen conceptual work and empirical studies reporting values greater than 0.90 as acceptable.

We first tested the measurement model. The measurement model included the following latent variables: satisfaction of the need for autonomy at time 1, satisfaction of the need for competence at time 1, satisfaction of the need for autonomy at time 2, satisfaction of the need for competence at time 2, epistemic curiosity at time 1, epistemic curiosity at time 2, and epistemic satisfaction at time 3. Results for the measurement model showed an acceptable model fit, χ^2^ = 497.37, *p* < 0.001 (df = 300), RMSEA = 0.06, CFI = 0.94, and TLI = 0.93. Examination of the standardized factor loadings showed that they were all significant and in the expected direction (ranging from 0.60 to 0.95). The latent correlations were of medium size with acceptable levels of discriminant validity (Brown 2006), ranging from 0.35 to 0.74. The h coefficients for each of the latent variables were acceptable: satisfaction of the need for autonomy at time 1 (0.83), satisfaction of the need for competence at time 1 (0.86), satisfaction of the need for autonomy at time 2 (0.87), satisfaction of the need for competence at time 2 (0.89), epistemic curiosity at time 1 (0.70), epistemic curiosity at time 2 (0.83), and epistemic satisfaction at time 3 (0.95). Given that the fit of the measurement model was acceptable, the structural model was tested. Yet, we need to point out that some fit indexes could have been higher to support the overall fit of the model (e.g., CFI and TLI ≥ 0.95).

**Figure 1:**
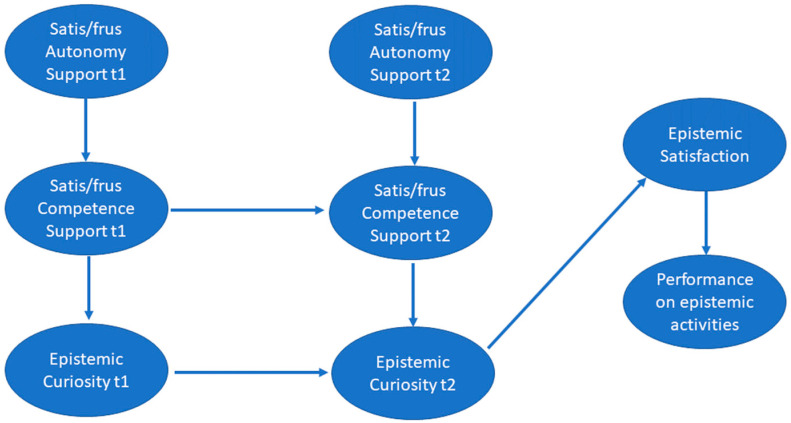
Conceptual propositions.

**Figure 2:**
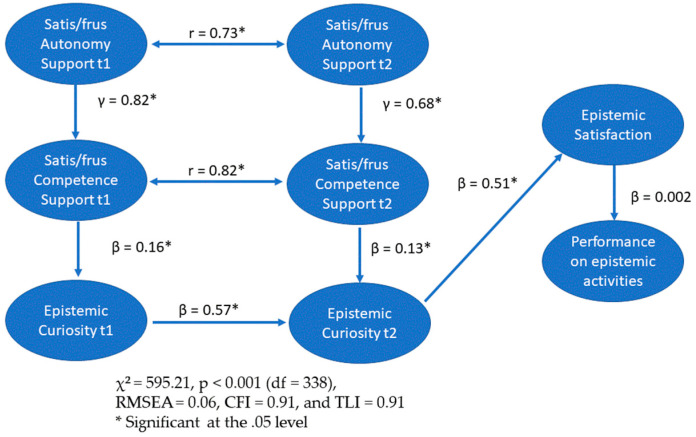
Summary of results.

Results for the structural model showed an acceptable model fit, χ^2^ = 595.21, *p* < 0.001 (df = 338), RMSEA = 0.06, CFI = 0.91, and TLI = 0.91 (see Table 1 for descriptive statistics and Figure 1 for conceptual propositions). Examination of the individual unstandardized coefficients with the 95% confidence interval showed significant relationships between time 1 and 2 satisfaction of the need for autonomy and satisfaction of the need for competence at time 1 and 2, γ = 0.82 (CI = 0.60, 1.14), *p* < 0.001 and γ = 0.68, (CI = 0.47, 0.98), *p* < 0.001, respectively. Satisfaction of the need for competence in times 1 and 2 was positively correlated with epistemic curiosity at times 1 and 2, β = 0.16, (CI = 0.09, 0.25), *p* < 0.001 and β = 0.13, (CI = 0.05, 0.23) *p* < 0.001. Whereas epistemic satisfaction at time 3 was positively correlated with epistemic curiosity at time 2, β = 0.51, (CI = 0.22, 0.89), *p* < 0.001, it was not significantly correlated with performance on epistemic activities, β = 0.03, (CI = −0.17, 0.33), *p* = 0.80. The indirect effects of autonomy time 1 on curiosity time 1, autonomy time 2 on curiosity time 2, autonomy time 2 on satisfaction, and autonomy time 2 on performance on epistemic activities were as follows: 0.243, (CI = 0.10, 0.38), *p* < 0.001, 0.218, (CI = 0.07, 0.37), *p* < 0.001, 0.099, (CI = 0.03, 0.17), *p* = 0.001, and 0.002, (CI = −0.02, 0.02), *p* = 0.80, respectively (see Table 2 and Figure 2 for summary of results). The coefficients of determination were satisfaction of the need for competence time 1 and 2 (0.44 and 0.31), epistemic curiosity time 1 and 2 (0.14 and 0.57), epistemic satisfaction (0.21), and performance on epistemic activities (0.0001), respectively.

## 5. Discussion

The purpose of the present investigation was to examine two antecedents of epistemic curiosity (satisfaction of the need for autonomy and competence) and two consequences of epistemic curiosity (epistemic satisfaction and performance on epistemic activities). Six hypotheses were tested and four were supported. The implications of the results were discussed.

### 5.1. Conceptual Implications

As students navigate their first attempts to generate ideas to seize opportunities for new business ventures, epistemic curiosity plays an important role. Epistemic curiosity is a driver of learning, a driver of lifelong learning needed in entrepreneurial education. Epistemic curiosity is characterized by liking the acquisition of new knowledge (Litman 2005), which could be facilitated by professors who satisfy students’ need for autonomy and competence. Our results first showed a positive relationship between the satisfaction of the need for autonomy and competence, supporting hypothesis one. These results are aligned with self-determination theory, which suggests that autonomy support helps to motivate and energize students´ behavior (Ryan and Deci 2018). Specifically, the satisfaction of the need for autonomy appeared to be a necessary condition for students to feel competent. An autonomy-supportive context can facilitate the acquisition of knowledge and skills, which in turn provide positive emotions such as satisfaction and well-being (Levesque et al. 2004). Consistent with the positive consequences of an autonomy support context, our results showed a positive indirect relationship between the satisfaction of the need for autonomy and epistemic curiosity, supporting hypothesis two. These results were consistent with the proposition that autonomy influences students´ interest in exploring and acquiring knowledge (Kashdan et al. 2004; Puerta-Sierra and Puente-Díaz 2023).

Regarding the satisfaction of the need for competence, our findings showed a positive direct relationship between competence satisfaction and epistemic curiosity, supporting hypothesis three. Our results were consistent with some of the main postulates of self-determination theory (SDT), which suggests that individuals are inherently curious to learn. This inherent curiosity is facilitated or hindered by the satisfaction of the need for competence (Ryan and Deci 2018). Our results were also consistent with empirical findings emphasizing the importance of the satisfaction of the need for competence in education (Wang et al. 2019). We posit that experiencing epistemic curiosity is an optimal outcome, given that the students need to immerse themselves in a knowledge experience in entrepreneurial education. If entrepreneurship is framed as a life skill, students will need to value the importance of constantly acquiring and updating their knowledge. Epistemic curiosity might be the fuel that energizes lifelong learning.

Our results also showed a positive relationship between epistemic curiosity and epistemic satisfaction, supporting hypothesis four. Similar to the study of Chukwuedo and Ogbuanya (2020), in which the authors found that an academic intervention can increase the students´ satisfaction and curiosity, our results showed a close relationship between epistemic curiosity and epistemic satisfaction coming from engaging in epistemic activities in the context of entrepreneurial education. These results were also consistent with the idea that epistemic emotions play an important role in education (Nerantzaki et al. 2021), where epistemic activities are part of the learning process (Puente-Díaz and Puerta-Sierra 2024).

Conversely, we did not find support for a positive indirect relationship between epistemic curiosity and performance on epistemic activities, failing to support hypothesis five. Despite the important role of a motivational supportive environment and the experience of positive emotions (Wang et al. 2019), our results were only able to show support for epistemic satisfaction and not for performance on epistemic activities. Similarly, we did not find support for a positive direct relationship between epistemic satisfaction and performance on epistemic activities, failing to support hypothesis six. Our results were consistent with the previous work of Blanz (2014), in which the author found no significant relationship between satisfaction and indicators of academic performance. One possible explanation is that performance on epistemic activities is energized and explained by multiple factors. Having an autonomy- and competence-supportive environment and experiencing epistemic curiosity might not be enough to significantly influence performance on epistemic activities.

In sum, our results provide evidence for the satisfaction of the need for autonomy and competence, as relevant sources of epistemic curiosity and epistemic satisfaction. The results have implications for teachers and academic institutions guiding entrepreneurship students during their process of experiential venture creation and opportunity identification.

### 5.2. Implications for Education

Our results have implications for education. As mentioned previously, planting the seed of curiosity in students is a key element for present and future academic or professional tasks and decisions. Hence, entrepreneurship educators could start by letting students experience more freedom (autonomy) to self-organize their activities (Levesque et al. 2004), with the purpose of increasing the students´ competence (the ability to do what they aim for) (Wang et al. 2019). Providing teachers with this perspective could help develop students´ entrepreneurial mindset with relevant implications for life-long learning. Previous studies in education have provided experimental evidence for the important role of the satisfaction of the need for autonomy and for how to enhance it by providing students with choices (e.g., Puerta-Sierra and Puente-Díaz 2023). A second implication is to continue using project-based methodologies in entrepreneurial education to plant the seed of curiosity. Learning sciences have shown that project-based methodologies lead to greater engagement, which can be seen as a behavioral indicator of curiosity (e.g., Blumenfeld et al. 2006).

### 5.3. Limitations

Our main goal was to show that satisfaction of the need for autonomy and competence are two relevant antecedents of epistemic curiosity, and epistemic satisfaction and performance on epistemic activities are two adaptive consequences of epistemic curiosity. Nevertheless, we found partial support for our propositions. Although autonomy and competence are associated with positive outcomes (Levesque et al. 2004) and enhance students´ motivation (Wang et al. 2019), which often leads to epistemic curiosity, in our study, this was not enough to have an impact on performance on epistemic activities. The lack of significant results could have two possible explanations: (1) One possibility is that satisfied students do not work better than unsatisfied ones, or that well-performing students are not more satisfied than lower-performing ones (Blanz 2014). (2) Another possibility is that our epistemic activities were not sensitive enough to capture individual differences in performance explained by epistemic curiosity and satisfaction.

Another limitation was that the entrepreneurship courses used for this study were (1) mandatory, and (2) the sample of entrepreneurship students consisted of students from different disciplines, including business, medicine, arts, and engineering, among others. The nature of the class and the heterogeneity of students could have impacted performance on epistemic activities beyond the variables assessed in our study.

### 5.4. Future Studies

We see several directions for future research coming from the lack of support for hypotheses five and six and from the current state of knowledge in the connection between the satisfaction of needs and epistemic curiosity in entrepreneurship education. (1) Future research should continue examining the relationship between epistemic curiosity and different indicators of academic performance within the entrepreneurship education environment. (2) Future studies could complement our results by incorporating other epistemic emotions, frustration, doubt, and pride, in specific steps during the entrepreneurial process and with different performance outcomes. (3) Given the nature of entrepreneurial education and active learning, future studies should use mixed-method designs in which personal interviews, observations, and ethnography are conducted in combination with observational quantitative studies. (4) While it is important to continue conducting research with entrepreneurship students, future studies should be conducted with actual entrepreneurs and examine how epistemic curiosity drives their knowledge acquisition efforts. (5) Our last suggestion for future research is to conduct more experiments manipulating the type of information, urgency of information, or any other variable connected with information and knowledge and to test how they influence the experience of epistemic curiosity and deprivation-type curiosity, which was only discussed but not tested in the current study.

In sum, our study provided empirical evidence for the relevant role of autonomy and competence satisfaction in experiencing epistemic curiosity and epistemic satisfaction. These findings can be valuable not only for entrepreneurship education programs but also for other disciplines seeking to induce students into a more active learning process. In this sense, future research can also link epistemic emotions and co-creation in an academic context due to its interest in understanding the outcomes of actively involving students in their academic environment (Dollinger et al. 2018).

## Figures and Tables

**Table 1:** Descriptive statistics.

	Mean	SD
Autonomy satisfaction time 1	3.77	2.70
Autonomy satisfaction time 2	2.87	3.20
Competence satisfaction time 1	5.32	2.59
Competence satisfaction time 2	5.28	2.82
Epistemic curiosity time 1	7.80	1.40
Epistemic curiosity time 2	7.95	1.51
Epistemic satisfaction time 3	8.93	1.25
Performance on epistemic activities	8.45	1.69

**Table 2:** Summary of results.

Effect	Coefficient	*p*-Value
Satisfaction of autonomy time 1 on satisfaction of competence time 1	0.82	*p* < .001
* Satisfaction of autonomy time 2 on satisfaction of competence time 2	0.68	*p* < .001
Satisfaction of competence time 1 on epistemic curiosity time 1	0.16	*p* < .001
* Satisfaction of competence time 2 on epistemic curiosity time 2	0.13	*p* < .001
Epistemic curiosity time 2 on epistemic satisfaction	0.51	*p* < .001
Epistemic satisfaction on performance on epistemic activities	0.002	*p* = .80

* While controlling for competence time 1 and curiosity time 1, respectively.

## Data Availability

The raw data supporting the conclusions of this article will be made available by the authors on request.

## References

[B1-jintelligence-12-00127] Berlyne Daniel Ellis (1954). A theory of human curiosity. British Journal of Psychology.

[B2-jintelligence-12-00127] Blanz Mathias (2014). How do study satisfaction and academic performance interrelate? An investigation with students of Social Work programs. European Journal of Social Work.

[B3-jintelligence-12-00127] Blumenfeld Phyllis C., Kempler Toni M., Krajcik Joseph S., Sawyer R. Keith (2006). Motivation and Cognitive Engagement in Learning Environments. The Cambridge Handbook of: The Learning Sciences.

[B4-jintelligence-12-00127] Brown Timothy A. (2006). Confirmatory Factor Analysis for Applied Research.

[B5-jintelligence-12-00127] Chukwuedo Samson O., Ogbuanya Chinyere T. (2020). Fostering Academic Major Satisfaction, Career Curiosity, and Job Search Behaviors Among Electrical/Electronic Technology Education Undergraduates. Journal of Career Development.

[B6-jintelligence-12-00127] De Sousa Ronald (2009). Epistemic feelings. Mind and Matter.

[B7-jintelligence-12-00127] Diener Ed (1984). Subjective well-being. Psychological Bulletin.

[B8-jintelligence-12-00127] Dollinger Mollie, Lodge Jason, Coates Hamish (2018). Co-creation in higher education: Towards a conceptual model. Journal of Marketing for Higher Education.

[B9-jintelligence-12-00127] Elnadi Moustafa, Gheith Mohamed Hani (2023). The role of individual characteristics in shaping digital entrepreneurial intention among university students: Evidence from Saudi Arabia. Thinking Skills and Creativity.

[B10-jintelligence-12-00127] Gruber Matthias J., Gelman Bernard D., Ranganath Charan (2014). States of curiosity modulate hippocampus-dependent learning via the dopaminergic circuit. Neuron.

[B11-jintelligence-12-00127] Haneberg Dag Håkon, Aaboen Lise, Middleton Karen W. (2022). Teaching and facilitating action-based entrepreneurship education: Addressing challenges towards a research agenda. The International Journal of Management Education.

[B12-jintelligence-12-00127] Heinemann Henrik, Mussel Patrick, Schäpers Philipp (2022). Curious enough to start up? How epistemic curiosity and entrepreneurial alertness influence entrepreneurship orientation and intention. Frontier in Psychology.

[B13-jintelligence-12-00127] Kashdan Todd B., Disabato David J., Goodman Fallon R., Doorley James D., McKnight Patrick E. (2020). Understanding psychological flexibility: A multimethod exploration of pursuing valued goals despite the presence of distress. Psychological Assessment.

[B14-jintelligence-12-00127] Kashdan Todd B., Rose Paul, Fincham Frank D. (2004). Curiosity and exploration: Facilitating positive subjective experiences and personal growth opportunities. Journal of Personality Assessment.

[B15-jintelligence-12-00127] Lackéus Martin (2020). Comparing the impact of three different experiential approaches to entrepreneurship in education. International Journal of Entrepreneurial Behavior.

[B16-jintelligence-12-00127] Levesque Chantal, Zuehlke A. Nicola, Stanek Layla R., Ryan Richard M. (2004). Autonomy and Competence in German and American University Students: A Comparative Study Based on Self-Determination Theory. Journal of Educational Psychology.

[B17-jintelligence-12-00127] Litman Jordan, Renninger K. Ann, Hidi Suzanne E. (2019). Curiosity: Nature, dimensionality, and determinants. The Cambridge Handbook of Motivation and Learning.

[B18-jintelligence-12-00127] Litman Jordan A. (2005). Curiosity and the pleasures of learning: Wanting and liking new information. Cognition and Emotion.

[B19-jintelligence-12-00127] Litman Jordan A., Spielberger Charles D. (2003). Measuring epistemic curiosity and its diversive and specific components. Journal of Personality Assessment.

[B20-jintelligence-12-00127] Morton Adam, Goldie Peter (2010). Epistemic emotions. The Oxford Handbook of Philosophy of Emotion.

[B21-jintelligence-12-00127] Muis Krista R., Chevrier Marianne, Singh Cara A. (2018). The role of epistemic emotions in personal epistemology and self-regulated learning. Educational Psychologist.

[B22-jintelligence-12-00127] Neck Heide M., Corbet Andrew C. (2018). The Scholarship of Teaching and Learning Entrepreneurship. Entrepreneurship Education and Pedagogy.

[B23-jintelligence-12-00127] Nerantzaki Katerina, Efklides Anastasia, Metallidou Panayiota (2021). Epistemic emotions: Cognitive underpinnings and relations with metacognitive feelings. New Ideas in Psychology.

[B24-jintelligence-12-00127] Nerantzaki Katerina, Efklides Anastasia (2019). Epistemic emotions: Interrelationships and changes during task processing. Hellenic Journal of Psychology.

[B25-jintelligence-12-00127] Pazos Pilar, Pérez-López María Carmen, González-López María José (2022). Examining teamwork competencies and team performance in experiential entrepreneurship education: Emergent intragroup conflict as a learning triggering event. Education + Training.

[B26-jintelligence-12-00127] Podsakoff Philip M., MacKenzie Scott B., Podsakoff Nathan P. (2012). Sources of method bias in social science research and recommendations on how to control it. Annual Review of Psychology.

[B27-jintelligence-12-00127] Puente-Díaz Rogelio (2023). Metacognitive feelings as a source of information for the creative process: A conceptual exploration. Journal of Intelligence.

[B28-jintelligence-12-00127] Puente-Díaz Rogelio, Puerta-Sierra Lizbeth (2024). Epistemic emotions in epistemic activities: The necessary yet insufficient condition to generate, evaluate, and select ideas. Thinking Skills and Creativity.

[B29-jintelligence-12-00127] Puente-Díaz Rogelio, Cavazos-Arroyo Judith, Puerta-Sierra Lizbeth (2024). The role of epistemic emotions and activities in creative action: A metacognitive and self-regulatory approach. The Journal of Creative Behavior.

[B30-jintelligence-12-00127] Puerta-Sierra Lizbeth, Puente-Díaz Rogelio (2023). Co-creation in entrepreneurship education: How autonomy support enhances the intention to develop entrepreneurial ideas. Journal of Education for Business.

[B31-jintelligence-12-00127] Ryan Richard M., Deci Edward L. (2018). Self-Determination Theory: Basic Psychological Needs in Motivation, Development, and Wellness.

[B32-jintelligence-12-00127] Syed Imran, Butler Jonathan Craig, Smith Ronda M., Cao Xian (2020). From entrepreneurial passion to entrepreneurial intentions: The role of entrepreneurial passion, innovativeness, and curiosity in driving entrepreneurial intentions. Personality and Individual Differences.

[B33-jintelligence-12-00127] Van der Kaap-Deeder Jolene, Soenens Bart, Ryan Richard M., Vansteenkiste Maarten (2020). Manual of the Basic Psychological Need Satisfaction and Frustration Scale (BPNSFS).

[B34-jintelligence-12-00127] Van der Kaap-Deeder Jolene, Vansteenkiste Maarten, Soenens Bart, Loeys Tom, Mabbe Elien, Gargurevich Rafael (2015). Autonomy-supportive parenting and autonomy-supportive sibling interactions: The role of mothers’ and siblings’ psychological need satisfaction. Personality and Social Psychology Bulletin.

[B35-jintelligence-12-00127] Wang C. K. John, Liu Woon C., Kee Ying H., Chian Lit K. (2019). Competence, autonomy, and relatedness in the classroom: Understanding students’ motivational processes using the self-determination theory. Heliyon.

[B36-jintelligence-12-00127] West Stephen G., Taylor Aaron B., Wu Wei, Hoyle Rick H. (2012). Model fit and model selection in structural equation modeling. Handbook of Structural Equation Modeling.

[B37-jintelligence-12-00127] Ye Qing, Zhou Rongting, Anwar Muhammad Azfar, Siddiquei Ahmad Nabeel, Hussain Siraj, Asmi Fahad (2022). Virtual reality-based learning through the lens of eudaemonic factors: Reflective thinking as a game changer. Thinking Skills and Creativity.

